# Influence of Common Non-Synonymous Toll-like Receptor 4 Polymorphisms on Bronchopulmonary Dysplasia and Prematurity in Human Infants

**DOI:** 10.1371/journal.pone.0031351

**Published:** 2012-02-14

**Authors:** Pascal M. Lavoie, Mihoko Ladd, Aaron F. Hirschfeld, Johanna Huusko, Mari Mahlman, David P. Speert, Mikko Hallman, Thierry Lacaze-Masmonteil, Stuart E. Turvey

**Affiliations:** 1 Child & Family Research Institute of British Columbia, Vancouver, Canada; 2 Division of Neonatology, Department of Pediatrics, University of British Columbia, Vancouver, Canada; 3 Division of Infectious and Immunological Diseases, Department of Pediatrics, University of British Columbia, Vancouver, Canada; 4 Oulu University Central Hospital and Department of Pediatrics, University of Oulu, Oulu, Finland; 5 Children's Hospital of Eastern Ontario, University of Ottawa, Ontario, Canada; Hôpital Robert Debré, France

## Abstract

Bronchopulmonary dysplasia (BPD) is a common chronic lung disease and major risk factor for severe respiratory syncytial virus (RSV) infection among preterm infants. The Toll-like receptor 4 (TLR4) is involved in oxidative injury responses in the lungs. Two non-synonymous single nucleotide polymorphisms in the *TLR4* gene have been associated with RSV infection in children. However, it is unclear to what extent this association is confounded by BPD or prematurity. In this study, we analyzed two population-based cohorts of preterm infants at risk for BPD as well as ethnicity-matched infants born at term, to test whether the TLR4 polymorphisms Asp299Gly (rs4986790) and Thr399Ile (rs4986791) are independently associated with BPD or premature birth. In a Canadian cohort (n = 269) composed of a majority of Caucasian preterm infants (BPD incidence of 38%), the *TLR4*-299 heterozygous genotype was significantly under-represented in infants without BPD (1.6% of infants versus 12% in infants with severe BPD) after adjusting for twins, ethnicity, gestational age, birth weight and gender (p = 0.014). This association was not replicated in a Finnish cohort (n = 434) of premature singletons or first-born siblings of Caucasian descent, although the incidence of BPD was substantially lower in this latter population (15%). We did not detect a significant association (>2-fold) between *TLR4* genotypes and prematurity (p>0.05). We conclude that these *TLR4* genotypes may have, at best, a modest influence on BPD severity in some populations of high-risk preterm infants. Further studies are warranted to clarify how clinical heterogeneity may impact genetic susceptibility to BPD.

## Introduction

Bronchopulmonary dysplasia (BPD) is a serious chronic inflammatory lung disease frequently observed in premature infants born at less than 32 weeks of gestation [1]. Children suffering from BPD have considerably increased risk of severe airway disease due to respiratory syncytial virus (RSV) infection. Nearly half of preterm infants with BPD require hospitalization due to RSV in the first year of life compared to fewer than 5% of term-born children less than 6 months of age [2], [3]. RSV is the most frequent cause of mortality due to respiratory infection in children under 2 years of age [4]. Passive immunoprophylaxis using palivizumab, a humanized monoclonal antibody directed against the F protein of RSV, effectively reduces disease severity in high-risk populations [5], [6]. However, its high cost limits its availability, therefore it is paramount to understand why some infants appear to be at increased risk for severe disease [7].

Genetic factors likely play an important role in determining susceptibility to respiratory disease such as BPD or RSV [8]. The innate immune Toll-like receptor 4 *(TLR4)* gene has been directly implicated in the immune response against RSV [9], [10]. Two common non-synonymous polymorphisms (minor allele frequency <3%) in the human *TLR4* gene (referred to as Asp299Gly and Thr399Ile based on the amino acid permutations they encode) are in strong linkage disequilibrium and potentially impact function of the receptor [11], [12]. Examination of a large group of premature infants with symptomatic RSV, including 41% of premature infants with BPD, showed that the frequency of double heterozygous *TLR4*-299/-399 genotypes was substantially greater (89.5%) compared to literature controls (∼10.5%) [13]. However, in children born at term, our group and others observed only a modest increase in these two *TLR4* genotypes (16% and 17%) in relation with severe RSV infection or no significant increase at all [14], [15], [16], [17]. Taken together, these data suggest that there may be a relatively large confounding association between the *TLR4*-299/-399 genotypes and BPD, or even prematurity.

BPD involves an arrested vascular and alveolar pulmonary development, exacerbated by inflammation and oxidative lung injury [1]. Recently, twin studies also demonstrated a strong heritability [18], [19]. Because genetic risk factors appear to play an important role in BPD and because this disease is the most important risk factor for RSV infection, a confounding genetic association is plausible. A potential modulation of the BPD severity due to functional variants in the *TLR4* gene is also consistent with an emerging appreciation of the role of TLR4 in protecting lung tissue against oxidative damage ([20], [21] and see discussion). In this study, we aim to determine whether there is an independent epidemiological association between *TLR4* polymorphisms and BPD in two cohorts of at-risk preterm infants.

## Results

### Association between *TLR4* genotypes and BPD

Preterm cohort A consisted of 269 preterm infants (mean gestational age: 27.0±1.8 weeks; mean birth weight: 1023±315 grams). Of these infants, 65% were of Caucasian origin (see complete ethnic distribution in **Table 1**) and 34% (n = 94) were twins including 72 concordant (same-sex) twins. The overall incidence of infants with moderate or severe BPD was 38% (n = 103 out of 269). Of the infants in preterm cohort A, 246 (91%) were alive at 36 weeks post-menstrual age (PMA). Causes of death before 36 weeks post-menstrual age (PMA) in infants in preterm cohort A included early severe intraventricular hemorrhage, necrotizing enterocolitis or sepsis; none of which were thought to be directly from BPD and therefore, these infants were excluded from the BPD outcome definition.

**Table 1:** Ethnicity of infants in preterm cohort A and matched control term infants.

Ethnicity, n (%)	Preterm cohort A (n = 269)	Term-born (n = 201)	95%CI for difference (%)†	Combined heterogenous/rare homozygous genotypes, n (%; 95%CI)*
Asian/Oriental	27 (10)	34 (17)	−13 to −0.5	NF
Black/African	6 (2.2)	4 (2.0)	−2.4 to 2.9	2 (20; 3 to 56)
Caucasian/European	176 (65)	145 (72)	−15 to 1.7	32 (10; 6.9 to 13)
First Nations	34 (13)	2 (1.0)	7.5 to 16	3 (10; 2 to 23)
Indian/Pakistani	10 (3.7)	8 (4.0)	−3.8 to 3.4	3 (20; 4 to 41)
Latin American	3 (1.1)	5 (2.5)	−3.8 to 1.1	NF
Middle Eastern	5 (1.9)	3 (1.5)	−2.0 to 2.7	NF
undeclared	8 (3.0)	0	1.0 to 5.0	1 (13; 0 to 53)

† Differences considered statistically significant are underlined.

* Combined heterogenous/rare homozygous genotype includes both preterm and infants born at term. NF  =  none found. The term “First Nations” refers to Canadian Indigenous Nations as defined by the Government of Canada.

In preterm cohort A, a majority of *TLR4*-299 heterozygous genotypes were observed in infants with moderate or severe BPD (60%; [95%CI: 41% to 85%]) compared to infants without BPD (i.e. defined as no oxygen at 28 days or 36 weeks; 5%; [95%CI: 0.01% to 25%]). The frequency of combined heterozygous/rare homozygous *TLR4*-299 genotype was greater in preterm neonates with moderate or severe BPD (n = 103) compared to preterm neonates without BPD (n = 62) used as a control group (12% versus 1.6%; p = 0.016; **Table 2**). When considering only neonates with severe BPD (n = 32), the frequency of either combined heterozygous/rare homozygous *TLR4*-299 or *TLR4*-399 genotypes were even higher (16%; p = 0.008 and p = 0.032, respectively). Only one preterm neonate was homozygous for the rare *TLR4*-299 allele; this infant had severe BPD and required prolonged mechanical ventilation for over 45 days. Altogether, these data indicate a potential genetic association between the two *TLR4*-299 and −399 genotypes and BPD severity.

**Table 2:** Association between *TLR4* genotypes and BPD in preterm cohort A.

Clinical characteristic	No BPD (n = 62)	Mild BPD (n = 81)	Moderate or Severe BPD (n = 103)	Death <36 weeks PMA (n = 23)	95%CI for difference§	p value§
GA, mean ± SD, wk	28.7±1.4	27.4±1.4	26.1±1.6	25±1.5	−3.1 to −2.1 †	
BW, mean ± SD, g	1301±244	1099±259	866±263	710±135	−516 to −354	
Gender, n (%) male	24 (39)	44 (54)	49 (48)	15 (65)	−6.6 to 24	
Ethnicity, n (%) Caucasian	44 (71)	50 (61)	66 (64)	14 (61)	−22 to 7.7	
Centre, n (%) from B	16 (26)	40 (49)	40 (39)	14 (61)	−1.4 to 27	
Genotype*						
TLR4-299, n (%)	1 (1.6)	7 (8.6)	12 (12)	2 (8.7)	3.1 to 17	0.016
TLR4-399, n (%)	2 (3.2)	6 (7.4)	12 (12)	2 (8.7)	0.8 to 16	0.06

GA: Gestational age; BW: Birth weight; 95%CI: 95% confidence interval.

* Combining heterozygous/rare homozygous genotypes.

† Statistically significant differences are underlined;

§ Comparing the Moderate/Severe with the No BPD group.

The association between the *TLR4* genotypes and BPD severity remained significant after adjusting for twins, gestational age, birth weight, gender, Caucasian origin and recruitment centre (p = 0.014; **Table 3**). The association also remained significant when adjusting for blood culture-positive post-natal sepsis, as well as for antenatal chorioamnionitis (data not shown). Clinical characteristics of preterm infants recruited in either of the two recruitment centres in cohort A were comparable except for a higher proportion with severe BPD from Centre A (**Table 4**). In order to control for population heterogeneity, we repeated the regression analysis using the same co-variables, but including only Caucasian children. In this analysis, we confirmed the association between *TLR4* genotype and BPD severity (p = 0.020 for both *TLR4*-299 and *TLR4*-399 genotypes).

**Table 3:** Multiple regression analysis between clinical co-variables and *TLR4-299* genotype* (dependent variable).

Co-variable	Adjusted OR [95%CI]	p value
Gestational age (wk)	1.12 [0.77 to 1.63]	0.56
Birth weight (g)	1.00 [1.00 to 1.00]	0.78
Gender (male or female)	0.95 [0.36 to 2.49]	0.92
Ethnicity, (Caucasian or not)	1.05 [0.39 to 2.81]	0.93
Recruitment Centre (A or B)	0.50 [0.19 to 1.32]	0.16
BPD (none, mild, moderate or severe)	2.15 [1.17 to 3.97]	0.014

* Results are shown for combined heterozygous/rare homozygous genotypes in binary regression. Significance was also comparable using all three genotypes (Asp/Asp, Asp/Gly and Gly/Gly) in regression models using BPD (p = 0.016) as the dependent variable.

**Table 4:** Clinical characteristics of infants in preterm cohort A.

Clinical characteristic	Centre 1 (n = 159)	Centre 2 (n = 110)	95%CI for difference†
Gestational age, mean ± SD, wk	27±1.8	27±1.8	−46 to 46
Birth weight, mean ± SD, g	1016±329	1033±294	−94 to 60
Male gender, n (%)	86 (54)	49 (45)	−2.6 to 22
Caucasian, n (%)	103 (65)	73 (66)	−13 to 10
Death prior to 36 wks PMA, n (%)	9 (5.7)	14 (13)	−14 to 0.1
BPD, n (%)*	63 (42)	40 (42)	−8.5 to 15
Severe BPD, n (%)*	25 (17)	7 (7.3)	2.1 to 17
Duration of supplemental oxygen, days (median, interquartile range)& ^,^ £	17 (1–62)	25 (3–56)	NC
Duration of mechanical ventilation, days (median, interquartile range)£	7 (1–27)	3 (1–17)	NC

& Up to transfer to peripheral centre or discharge home.

* Based on neonates alive at 36 weeks PMA.

† Differences considered statistically significant are underlined.

£ Differences were not statistically significant (p>0.05) based on non-parametric Mann-Whitney U testing. NC = Not calculated.

### Replication of the *TLR4* genotype association with BPD

In order to replicate the TLR4-BPD association detected in Preterm cohort A, we examined another population-based cohort of Finnish infants. Preterm cohort B consisted of 434 singletons or first-born sibling infants alive at 36 weeks PMA (mean gestational age: 29.5±2.1 weeks; mean birth weight: 1268±375 grams). The incidence of infants with BPD requiring supplemental oxygen at 36 weeks PMA was generally low (15%). In these infants, the proportions of genotypes in infants with or without BPD were similar and we did not detect a similar association between either *TLR4*-299 and −399 genotypes and BPD severity (**Table 5**).

**Table 5:** Association between *TLR4* genotypes and BPD in preterm cohort B.

Clinical characteristic	No BPD (n = 311)	Mild BPD (n = 56)	Moderate BPD (n = 52)	Severe BPD (n = 15)	p value†
GA, mean ± SD, wk	30.1±1.6	27.4±1.8	28.2±2.2	28.3±2.0	
BW, mean ± SD, g	1377±344	960±265	1008±327	988±307	
Gender, % male	59	46	65	80	
Genotype*					
TLR4-299, n (%)	61 (20)	10 (18)	16 (31)	3 (20)	0.31
TLR4-399, n (%)	61 (20)	10 (18)	16 (31)	3 (20)	0.31

GA: Gestational age; BW: Birth weight;

* Combining heterozygous/rare homozygous genotypes.

† Chi-square or Fisher exact as indicated.

### *TLR4* genotype and prematurity

In order to examine potential associations between *TLR4* genotypes and prematurity in preterm cohort A, infants were compared to a control group of term-born neonates matched by self-declared maternal ethnicity. As expected, both *TLR4*-299 and −399 heterozygous variants highly co-segregated (>99% of cases) in neonates from both cohorts (data not shown). The overall frequencies of the heterozygous *TLR4*-299 and −399 genotypes in both term-born cohort (9.5 and 9.0, respectively) and preterm cohort A (7.8 and 8.2, respectively) were consistent with frequencies previously reported in healthy children [13], indicating negligible selection bias in either groups. There was no significant difference between either *TLR4*-299 or −399 genotype frequencies in preterm cohort A and term-born infants (p>0.05). Moreover, in regression analysis we did not detect associations between gestational age, birth weight, gender or ethnicity, and either combined −299- or −399 heterozygous/rare homozygous genotype within the group of preterm neonates (p>0.05).

## Discussion

In this study, we specifically addressed the influence of two common non-synonymous *TLR4* gene polymorphisms on BPD and prematurity. An influence of *TLR4*-299/-399 genotypes on BPD severity is highly biologically plausible given the major role of this receptor in lung protection against the effects of hyperoxia and compelling body of evidence indicating that these genotypes may alter TLR4 responsiveness [20], [21]. TLR4 is expressed by alveolar epithelial cells [22]. Genome-wide linkage analyses have identified this gene as a main determinant of oxidative lung injury in mice [23]. In animal models, TLR4-deficient mice show exaggerated susceptibility to a lethal hyperoxic lung insult [24], whereas mice with an induced TLR4 expression demonstrate resistance to hyperoxia [25]. Using population-based cohorts to minimize selection bias, we found a significant association between BPD and the *TLR4*-299 and −399 genotypes in preterm infants born ≤30 weeks of gestation recruited in a Canadian population of mixed ethnicity. However, this association was not replicated in a second Finnish cohort of singletons or first-born sibling preterm infants born <32 weeks of gestation recruited from a homogenous Caucasian population. In addition, a >2-fold association between extreme prematurity and the two *TLR4* polymorphisms was not detected.

Although *TLR4*-299/-399 genotypes appear to have little functional effect on peripheral blood innate immune functions [26], apical cell surface expression of the TLR4-299Gly and 399Ile variants was shown to be markedly reduced in primary respiratory epithelial cells [11], [27]. Functionally, the *TLR4*-299Gly and 399Ile allelic variants additively impair interaction with the co-receptors MD-2 and/or CD14; consistent with our genetic association data the minor −299Gly allele is reported to have a greater functional impact than the minor 399Ile allele [12]. In the case of preterm infants, the heterozygous *TLR4* genotype is more likely to reduce tolerance to excessive oxygen exposure in primary respiratory tissues, therefore predisposing some infants to BPD. Consistent with this mechanism, we found that the combined frequency of the heterozygous and rare homozygous *TLR4* genotypes was low in infants without BPD (1.2%) compared to the term-born infants or to previous reports in pediatric cohorts [15], [16], [26], which may imply a protective role of against BPD in some populations of at-risk preterm infants.

Replication of findings in independent populations is a widely accepted method to exclude false-positive associations in genetic association studies [28]. Nonetheless, given the relatively modest statistical significance we cannot exclude that the association with BPD observed in preterm cohort A is due to chance. Because the *TLR4* genetic association with BPD is preserved in our sensitivity analysis of the sub-group of Caucasian in preterm cohort A, lack of power or differences in the ethnic distribution are unlikely to explain the differences in association between preterm cohort A and B. Alternatively, variations in clinical practice (e.g. reduced utilization of supplemental oxygen, increase use of non-invasive ventilation, lower incidence of sepsis, etc.), which can be substantial among neonatal centers, may modulate genetic influences on BPD [29], a perspective that has not been sufficiently addressed to date [30]. Because the potential role of the candidate TLR4 variants in BPD is so biologically compelling and the association is robust after correction for covariables, we believe the latter possibility is likely. In this regard, it is noteworthy that the incidence of BPD and especially of severe BPD, is generally much lower in several other European countries, including Finland (i.e. cohort B), and this may mask potential genetic effects [31]. In order to clarify these questions, gene-environment analyses comparing outcomes in sizeable population-based cohorts differing in clinical practices or in the incidence of BPD is warranted.

Awomoyi and colleagues reported an association between RSV and *TLR4* genotypes of unusually high magnitude (odds ratios of 72.7; 95%CI [38.8 to 136]) [13]. Because a major proportion of infants in this study were born preterm at high risk of BPD, these two latter factors may have biased this association. Although we have not directly examined the outcome of RSV disease, our study is still important to clarify a potential large influence from these factors. The relatively modest effect size of the combined heterozygous/rare homozygous genotypes we detect in infants of the preterm cohort A with severe BPD (12%), is unlikely to be sufficient to explain the strong over representation (89.5%) reported by Awomoyi [13]. Additional studies are required to address whether modest associations between *TLR4* polymorphisms and symptomatic RSV infections exist in children with or without BPD.

With regard to a possible modest association between *TLR4* variants and prematurity, slight enrichment in heterozygous/rare homozygous *TLR4*-299 genotype frequencies (from 16 to 23%) has been reported by Lorenz *et al.* in mid-preterm Finnish infants (i.e. 33±3.5 weeks of gestation) compared to infants born at term. More significant associations have not been detected in African infants born earlier in gestation (<33 weeks) [32], [33]. We found no association between prematurity and *TLR4*-299Gly and -399Ile alleles, however, it is important to appreciate that our cohort was not specifically powered to detect the small difference in *TLR4*-299 genotype frequency reported by Lorenz *et al.* [32]. Therefore, while our results are not inconsistent with these previous data, we exclude associations between the *TLR4*-299/-399 polymorphisms and prematurity, of magnitude greater than 2-fold.

In conclusion, our data support a potential modest influence of common non-synonymous variants in the biologically-compelling candidate gene *TLR4* on BPD, in some populations of at-risk preterm infants. Further studies are required to substantiate this finding and/or to clarify how variations in clinical practices among neonatal centers may influence genetic effects of *TLR4* polymorphisms on BPD severity in preterm infants.

## Methods

### Study populations and method of recruitment

This study was conducted according to the principles expressed in the Declaration of Helsinki. Parental written informed consent was obtained for all subjects recruited in this study. This study was approved by the University of British Columbia Clinical Research Ethics Board, the University of Alberta Ethics Board and the Research Ethics Board of Oulu University Hospital.

*Preterm cohort A:* The first preterm cohort consist of premature neonates of mixed ethnicity (see **Table 1** for a complete ethnic distribution) born at ≤30 completed weeks of gestation, sequentially recruited between June 2006 and June 2008, following admission to the neonatal intensive care unit of two large regional referral centres: the Children's & Women's Health (C&W) Centre of British Columbia (Vancouver, British Columbia, Canada) and the Royal Alexandra Hospital (RAH, Edmonton, Alberta, Canada) at birth. *Preterm cohort B:* The second preterm cohort consisted of neonates born <32 weeks of gestation. These were either singletons or, in cases of multiple gestations, the first-born sibling of first degree Finnish (a rather homogenous Caucasian population) maternal and parental descent sequentially recruited at the University Hospital of Oulu (Finland). These infants represent 80% of all very preterm infants from the district of Northern Finland. In both cohorts, only infants without major congenital anomalies of the cardiorespiratory system in whom written parental consent could be obtained were enrolled. Approximately 62%, 51% and 65% of eligible preterm infants in study period were enrolled at C&W, RAH or Oulu hospitals, respectively. Controls for the preterm cohort A were term-born infants (>37 weeks gestation) sequentially recruited at birth (C&W), from mothers undergoing elective repeat caesarean section deliveries in the context of a healthy pregnancy between February 2006 and August 2007.

### Clinical outcomes

Standard outcome measures that were the same between the two populations were used to limit the possibility of misclassification. BPD was defined as a chronic need for supplemental oxygen at 36 weeks of post-menstrual age (PMA) or at time of discharge home whichever came first, and further graded by severity using criteria adapted from the National Institute of Child Health and Human Development (NICHD) [18]. Respiratory outcome data were retrieved from the referral institution (up to discharge of the infant home) for all preterm neonates transferred before 36 weeks PMA, if on supplemental oxygen therapy and/or respiratory support for less than 72 h prior to transfer out of the main study centres. Preterm infants with BPD of moderate to severe grades were grouped together and compared to preterm infants without BPD on the basis that adverse and beneficial genetic variants would be enriched, respectively, in these two groups of disease severity. Blood-culture positive microbial sepsis in preterm infants was defined by clinical signs (e.g. temperature instability, cardiorespiratory instability, intestinal intolerance, etc.) accompanied with a single positive blood or cerebrospinal culture. Chorioamnionitis was defined following examination of placental histology, as a maternal stage 2 or greater including involvement of fetal membranes, and according to an established standard classification [34].

### Genotyping

DNA was either obtained from cord blood, umbilical cord tissue or peripheral blood depending on the circumstances of enrolment. DNA extraction was carried out using the ChargeSwitch gDNA Mini Tissue kit (Invitrogen) or the QIAamp DNA Blood Midi kit (QIAgen). Genotyping of *TLR4*-299 (reference dbSNP: rs4986790) and −399 (reference dbSNP: rs4986791) allelic variants was performed by real time quantitative PCR as previously described [15]. Genotype frequencies were in Hardy-Weinberg equilibrium (p>0.05 by chi-square test).

### Statistical analyses

In order to exclude a greater than 2-fold association between *TLR4*-299/-399 variants and BPD, we estimated that 200 preterm neonates in cohort A (estimated incidence of BPD of 35%) and 350 preterm neonates in cohort B (due to a lower estimated incidence of BPD of 20%) would be sufficient considering the ∼10% baseline *TLR4* genotype frequencies reported in the general population (power 80%; alpha error 5%). Similarly, we estimated that 200 term-born control neonates would allow us to detect at least a 2-fold difference in heterozygous allele frequencies between preterm and term-born infants. To detect an association with prematurity in preterm cohort A, infants were matched in frequency with term-born neonates according to self-reported maternal ethnicity. The Fisher exact or chi-square probability test were used to determine significance of differences in proportions, allelic or genotype frequency between groups. Multivariate regression analyses were performed using genotype or BPD as the dependant variable and included cited adjustment co-variables. For the preterm cohort A, regression models were also adjusted for twins using a co-variable defining each singleton or twin pair. 95% confidence intervals were calculated using the exact method. Statistics were computed using SPSS version 11.0 for Microsoft Windows.
